# Orthodontic treatment and biological limits: a retrospective clinical trial

**DOI:** 10.1186/s13005-023-00399-6

**Published:** 2023-12-14

**Authors:** Niki Nikoleta Tabancis, Karl-Friedrich Krey, Franka Stahl, Valeria Behnke, Anja Ratzmann

**Affiliations:** 1 Department of Orthodontics and Craniofacial Orthopaedics, University Medicine of Greifswald, Walther-Rathenau-Straße 42a, 17489 Greifswald, Germany; 2 Department of Orthodontics and Craniofacial Orthopaedics, University Medicine of Rostock, Strempelstraße 13, 18057 Rostock, Germany

**Keywords:** Treatment outcome, 6-PTV, 1-NB [mm], WALA ridge, Biological limits

## Abstract

**Background:**

The fundamental part of every successful orthodontic treatment is the detailed treatment planning including a precise determination of the virtual treatment objective (VTO) while considering the biological and anatomical limits. The aim of this study is to investigate and to compare the feasibility of the established reference values before and after orthodontic treatment and to determine the usefulness of this parameters as guidance for the sagittal anterior, sagittal posterior and transverse biological boundaries.

**Materials and methods:**

Thirty-two patients aged 9 to 18 years (12 male and 20 female) with all permanent teeth present were randomly selected for orthodontic treatment with fixed multibracket appliance regardless of the potential malocclusion. The parameters 6-PTV, 1-NB [mm] and the WALA ridge were set for the identification of the transverse, sagittal anterior and sagittal posterior tooth position. The measurements were carried out at the beginning (T0) and at the end (T1) of the orthodontic treatment. They were set in relation with their individual threshold values (G). After the results of the measurements were conducted using the software OnyxCeph3TM (version 3.2.185 (505), Image Instruments GmbH, Chemnitz, DE), they were statistically calculated in the software RStudio (2022.12.0 Build 353 © 2009–2022 Posit Software PBC).

**Results:**

Among the 32 patients, the mean pre- and post-treatment changes measured through the three parameters in relation to the individual reference values were statistically significant (*p* < 0.01). The mean values for 6-PTV, 1-NB and the WALA ridge amounted 15.37 mm, 2.56 mm and 4.23 mm at the beginning of the treatment, while after the treatment the measured values amounted 20.31 mm, 2.4 mm and 5.55 mm. These measurements combined with the statistical analysis of the changes of WALA ridge (T0, T1) confirmed that the teeth have been successfully uprighted and aligned. Furthermore, the maxillary first molars have been moved slightly mesially, as proven by the changes in 6-PTV, without certainty as to whether bodily movement or mesial tipping took place. Additionally, the lower incisors have been protruded, slightly exceeding the individual threshold values.

**Conclusion:**

The parameters investigated provide a suitable assessment tool for recording the limits of the sagittal posterior, the sagittal anterior and the transverse dimension.

## Introduction

As McNamara [1] stated, accurate orthodontic treatment is based on the previous detailed and systematic treatment planning. In the course of modernization, orthodontics is constantly making progress and is changing in its treatment planning and implementation, as many steps are carried out digitally. Given the multitude of contemporary tools of analysis and treatment alternatives, the existence of certain reference values is helpful in planning and achieving realistic and biologically acceptable treatment goals. In a previously published study, a literature research was conducted the parameters that could be significant for the orthodontic treatment. The following parameters were used as biological limits for the present investigation [2]: the WALA ridge [mm] in the mandible transverse dimension, the 6-PTV [mm] value in the sagittal dimension for the maxillary retromolar region and the 1-NB [mm] in the sagittal dimension for the lower incisors.

Hereby, 6-PTV [mm] is generally measured using a lateral cephalogram. It describes the distance between the distal occlusal contact point of the maxillary first molar (tooth 6) and the intersection of the pterygoid vertical (PTV) and thus detects the available retromolar space. This is an important information for evaluating the existing space for possible molar distalization [3, 4]. It is also helpful for detecting the eruption space of the third molars [5, 6]. In general, distalization of maxillary molars is a common method for gaining space that was lost because of the mesial movement of the maxillary posterior teeth [7]. Ricketts [3] collected digitally captured data and made it feasible to set a natural statistical distribution, taking into consideration growth and other influencing factors for the calculation of the retromolar space. With this intent, the vertical and the sagittal position of the maxillary first molars was measured through a computerized system and was set in relation to the Frankfort horizontal line and the pterygoid vertical line. The resulting equation is outlined as: Age + 3 mm = 6-PTV and is utilized in the present study for calculating the threshold value of the necessary retromolar space for each patient individually.

Steiner [8] emphasized the importance of anterior tooth position in relation to the soft tissues for a stable treatment result. This is also confirmed in further literature research [9, 10]. Hasund and Boö [11] analyzed the parameter 1-NB [mm] of 74 adults (37 male and 37 female) with an Angle class I, with harmonious soft tissue relation and a neutral profile without orthodontic treatment and compared the measurements with the equation regression set by Steiner (Y _1-NB [mm]_ = 0,25 * x _ANB_ + 3,5). The investigations aimed on incorporating two more variables (ML-NL and the N angle in combination with ANB). It is used as an expansion of Steiners’s linear equation by integrating augmented guiding variables to formulate a multiple linear equation (Y_1-NB [mm]_ = a_3_* x_ANB_ + a_2_*x_ML-NL_ + a_1_*x_N-angle_ + a_0_). 1-NB [mm] refers to a distance measured in a lateral cephalogram. It is located between the labialmost point of the lower incisors (tooth 1) and the vertical line from the nasion to the B-point, which is a reference measure point located on the anterior innermost convexity of the mandible between the alveolar junction and the pogonion.

Observing that this regression equation not only presented itself as more complex but also provide lower accuracy, they carried out additional research a few years later [12]. Assessing the inclination of the lower incisors is crucial for the orthodontic treatment planning. In this context it is useful to set the incisors inclination in relation to a vertical basal variable. The index (reference line between nasion and pogonion) in this case is not an advantage as it is very variable. The correlation coefficients are adversely influenced by the index, and the precision of the estimation with standard errors is imprecise as a five-percentage point smaller index results in a 0,4 mm more anteriorly position of the incisors. Segner and Hasund ascertain the potential of using the ANB angle and the distance between the vertical line from the pogonion to the nasion and the labialmost point of the lower incisors for and equation regression including the growth and the impact growth may have on the values used by a multiplier. The updated equation regression of Hasund and Segner is based on data from investigations they conducted on 275 orthodontically untreated young adults with an ideal occlusion originated from Hamburg, Munich and Bergen.

The equation regression of Steiner 1-NB [mm] = 0.51 * ANB—0.30 * Pg-NB [mm]—0.084 Index + 10.4 was replaced with the equation regression 1-NB [mm] = 0.50 * ANB—0.35 * Pg-NB [mm] + 3.9 to maximize accuracy. Ultimately, all other diagnostic measures and clinical observations should be compared with the calculated valued for the final determination of the treatment planning [12].

According to this regression equation, the analysis of lateral cephalogram and the analysis of dental casts have been merged in order to identify the ideal tooth position for the participants of the present study. That fusion contributes to the establishment of individual reference values, which limit lingual and labial movement of mandibular incisors.

The third parameter included in the present investigations is the WALA ridge. 1994 [13] William Andrews and Larry Andrews (WALA) established this anatomical line as an essential key to ideal occlusion and provide a reference line for determining an individual basal bone shape. They examined 1150 orthodontically treated and 120 orthodontically untreated patients and elucidated the occlusal differences between them [13] Moreover, the WALA ridge serves as anatomical reference line for limiting the amount of angulation and the buccolingual inclination of the dentition in the transverse dimension [14]. The WALA ridge defines an anatomical area which is described as the maximum contour line of the alveolar process in the transverse dimension. The distance between this maximum contour line and the dental facial axis-point (FA point), which lies in the most prominent point on the buccal surface of each tooth, mark the transverse limit implying the ideal position of mandibular teeth within this plane [14, 15]. Andrews further elaborated on the investigations measuring the distance between the WALA ridge and the FA point for all teeth. Their intention was to support the individualized orthodontic treatment planning and gives the opportunity to calculate to what extent teeth may be moved more buccally or lingually [16].

For ideal arch shape, the distance between the FA points of the according teeth and the WALA ridge should range between 0.1 mm and 2.2 mm, depending on the dental region [13, 16]. A few years later these results were confirmed through investigations of 65 orthodontically untreated patients [17].

The aim of the present study is the evaluation of the feasibility as well as the limits of certain tooth movements by focusing on the individual parameters described above. The main idea is to get away from creating simple digital setups, but rather generating virtual treatment objectives (VTO) with regard to the biological and anatomical limits in order to perform a responsible treatment.

## Material and methods

### Sample

**Fig. 1:**
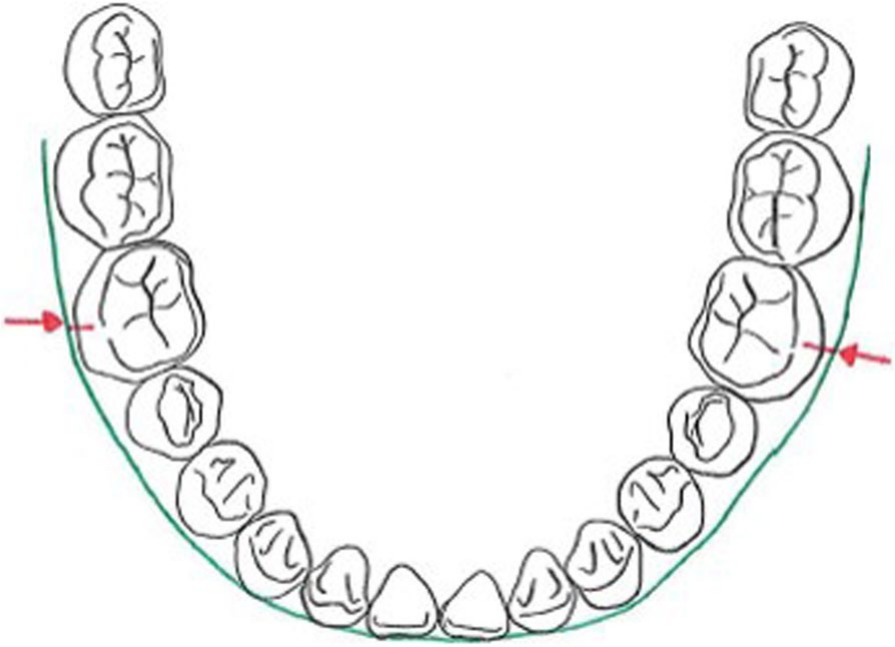
WALA ridge [mm]

**Fig. 2:**
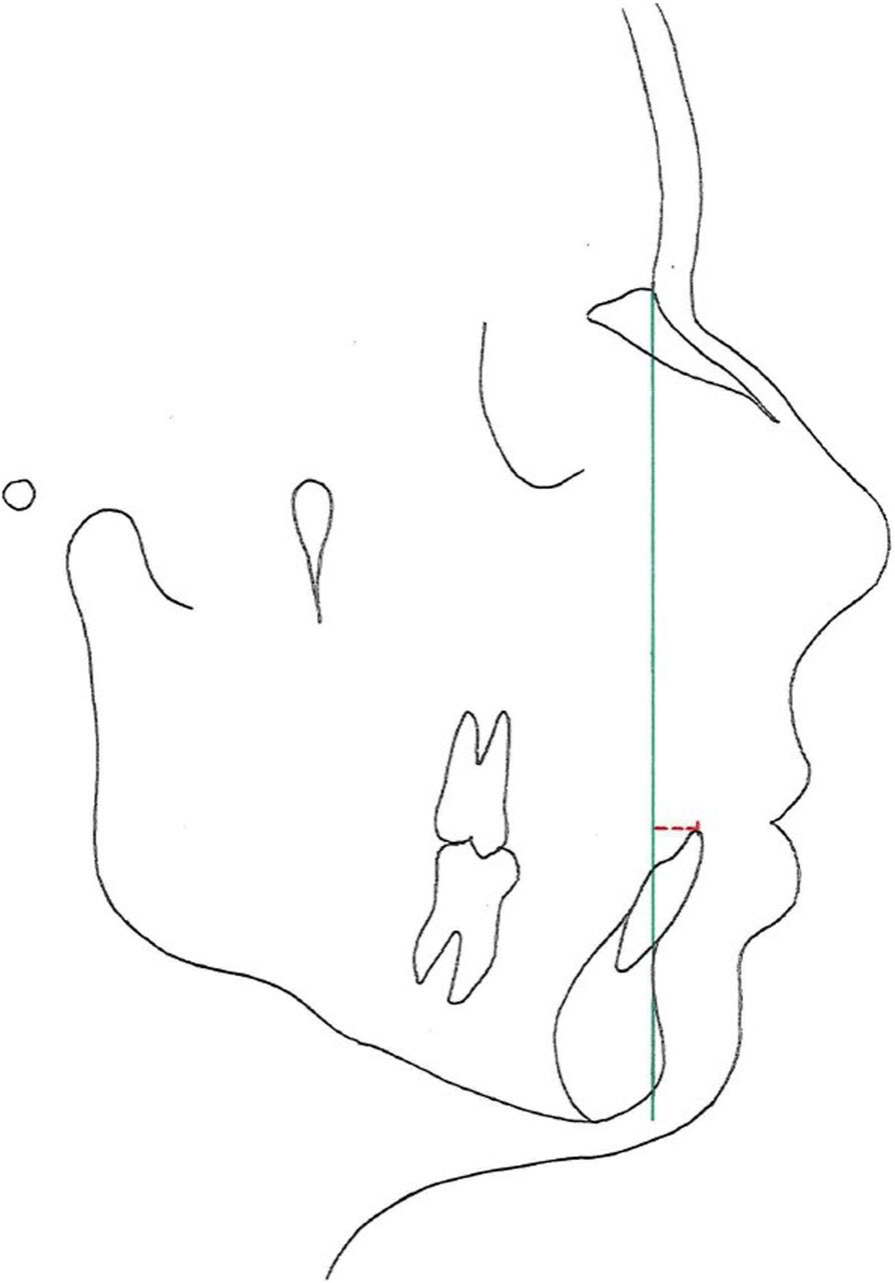
1-NB [mm]

**Fig. 3:**
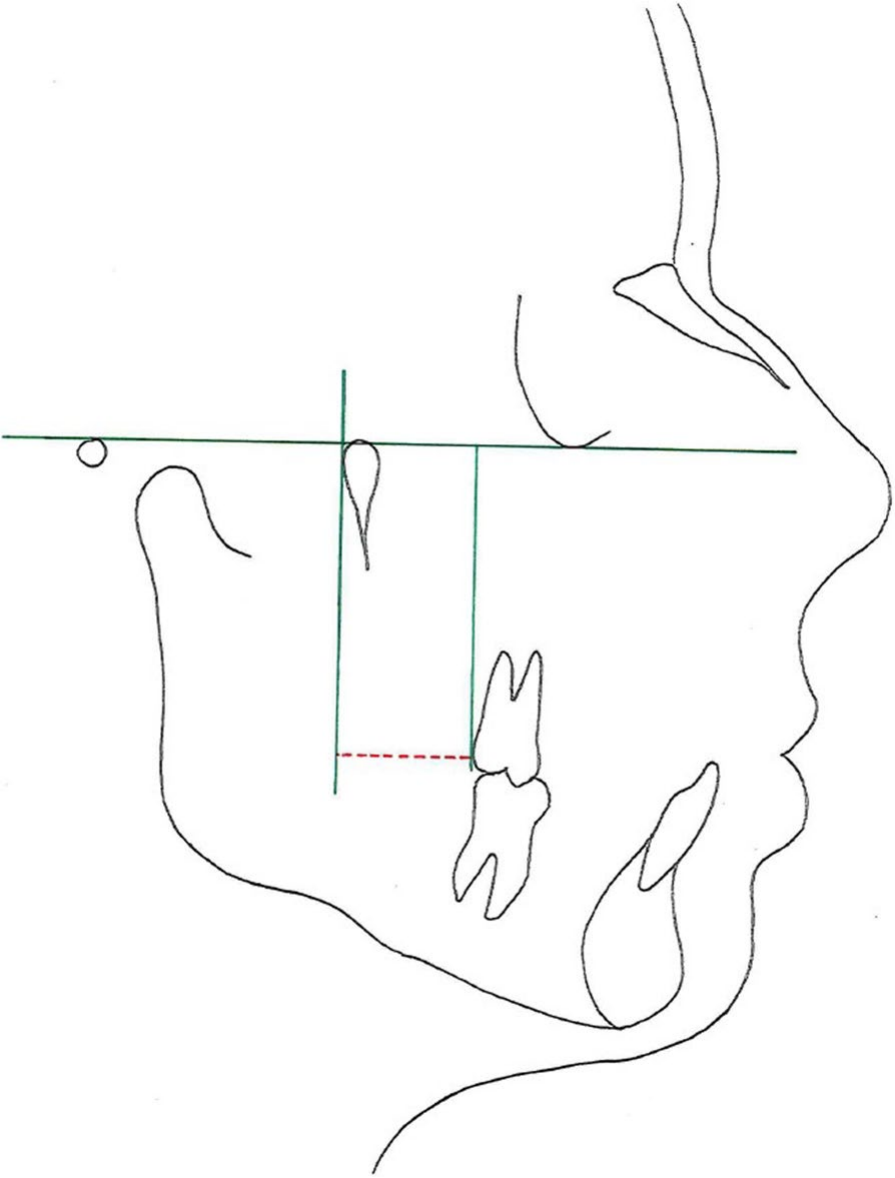
6-PTV [mm]

 In order to meet the requirements of this retrospective investigation, 32 participants were consecutively selected and fulfill the following inclusion criteria: (1) aged 9 to 16 years at the beginning, (2) having all permanent teeth fully erupted (excluding wisdom teeth), (3) good oral hygiene, (4) no prior history of orthodontic treatment, (5) no syndrome or severe dentofacial anomalies, (6) no systemic disease, (7) ongoing treatment with fixed appliance with 0.022 inch bracket slot and the torque and angulation values according to the treatment concept of R. P. McLaughlin, J. C. Bennett and H. J. Trevisi [18]. Furthermore, particular malocclusions were not subject to limitations. subjects consisted of 12 male and 20 female participants and were treated with a fixed multibracket appliance (Table 1). For the examination, the following three parameters were investigated: 6-PTV [mm], 1-NB [mm] and the WALA ridge [mm] (Figs. 1, 2 and 3). The main research objective focuses on investigating the eligibility of these three parameters as biological limits in the different planes of three-dimensional space within which the practitioner is able to realize safe orthodontic tooth movement.

**Table 1:** Sample description

Sample size (n)	age					
	T0			T1		
	min	max	mean	min	max	mean
**32 (all)**	9	16	13.52	11	18	16
**21 (female)**	9	16	12.54	11	18	15.1
**11 (male)**	11	15	13.38	14	18	15.5

### Sample size

The two-sample t-test power calculation showed that a minimum sample size of 16.7 patients was required, with regard to a confidence level of 0.95 and 80% power, to detect the differences between the measurements.

### Primary outcome

Initially, dental casts were digitalized with the 3D scanner Zirkonzahn Scanner S600 Arti (Zirkonzahn GmbH) in order to obtain 3D dental casts. The raw scans were processed and prepared for metric analysis in the OnyxCeph^3^™ software (module: “cast adjust”, module: “segmentation”) twice within a 30-day interval by a single investigator (N.N.T.). The investigator has undergone five years of specialized orthodontic training, which provides to measurement consistency and reliability. The WALA ridge was measured using the 3D dental casts in the OnyxCeph^3^™ software (module: “inspect 3D”). Additionally, lateral cephalograms were taken under standardized conditions (Philipps Bucky diagnost FS-TH and Dentsply Sirona Orthophos SL) and the parameters 6-PTV [mm] and 1-NB [mm] were measured and traced using the OnyxCeph^3^™ software (module: “analyze”).

### Statistical analysis

The statistical part includes a two-paired t-test and the Shapiro–Wilk test for the measurements of the three given parameters (6-PTV, 1NB [mm], WALA ridge) to assess the data distribution. Random error detection was implemented by Bland-Altmann analysis and intraclass correlation coefficient (ICC) was performed to verify the reproducibility of measurements [19–22]. The ICC is a proven method for determining the reliability of measurements and is recommended alongside the Bland–Altman plot [23, 24]. It was used at T0 (initial diagnosis).

All data were evaluated using the software RStudio (2022.12.0 Build 353 © 2009–2022 Posit Software PBC) [25]. The average values, the percentages and 95% confidence intervals (CI) of the measurements were calculated. The significance level, also denoted as alpha level, is the probability of rejecting the null hypothesis when it is true. In this study it was set at 0.05 for all parameters.

Since the data were normally distributed, the ANOVA and the Tukey test were utilized for the determination of differences between the patients [26, 27]. The measurements were compared before (T0) and after treatment (T1).

### Expected outcomes

The expected outcomes allow to cast an optimistic gaze on the course of the orthodontic treatment. Furthermore, it is assumed that the investigated values will remain within the biologically justifiable limits through the orthodontic treatment.

## Results

The data quality was evaluated through Bland–Altman analysis and the results are presented in Table 2. There was no statistically significant systematic error for the measurements of the three parameters. This confirms that the values obtained for 1-NB [mm] and the WALA ridge [mm] show a very high level of agreement in the measurements performed twice by a single orthodontist.

**Table 2:** Bland–Altman (LoA: Limit of Agreement, SD: standard deviation)

Parameter	Mean difference (bias)	bias SD	Upper LoA	Lower LoA
6-PTV	0.14375	0.7312991	1.577.096	-1.289596
WALA	0.003225806	0.2283767	0.4508441	-0.4443925
1-NBmm	-0.034375	0.2417802	0.4395142	-0.5082642

**Fig. 4:**
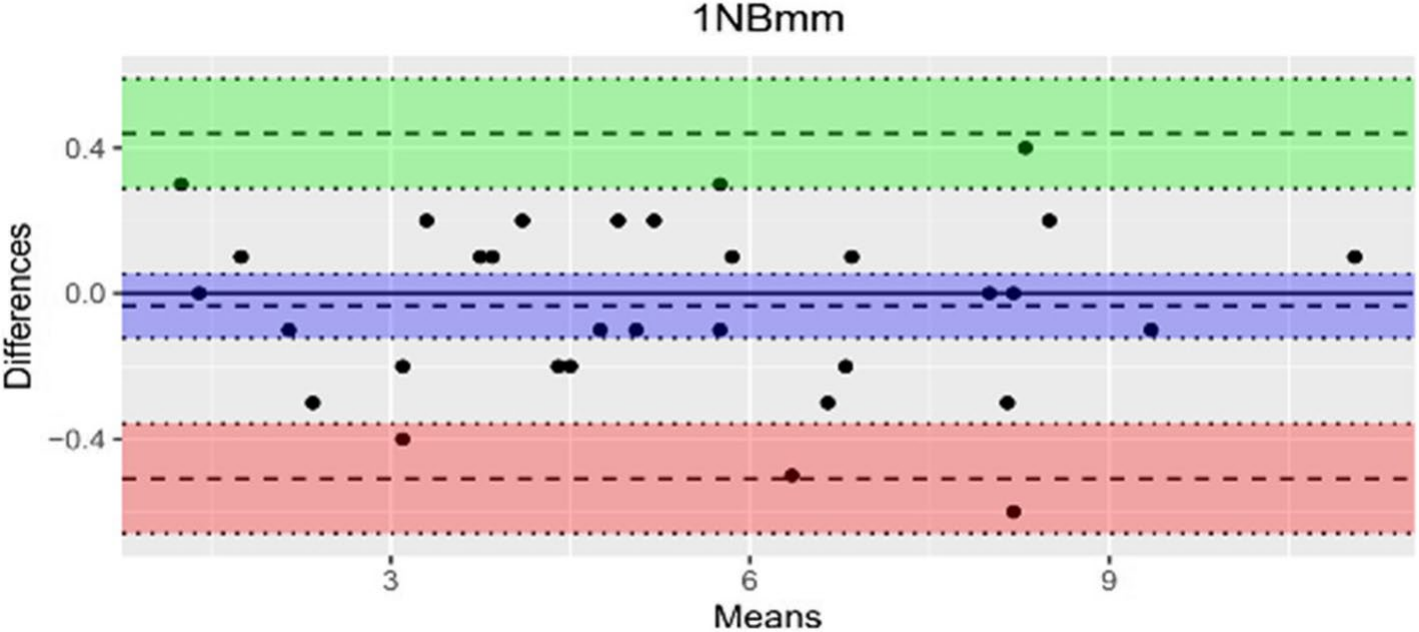
Bland-Altman 1-NB [mm]

**Fig. 5:**
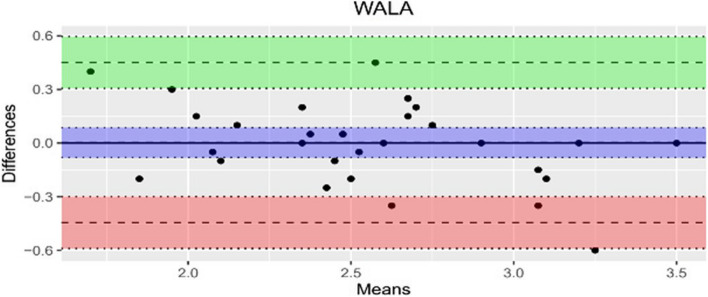
Bland-Altman WALA [mm]

**Fig. 6:**
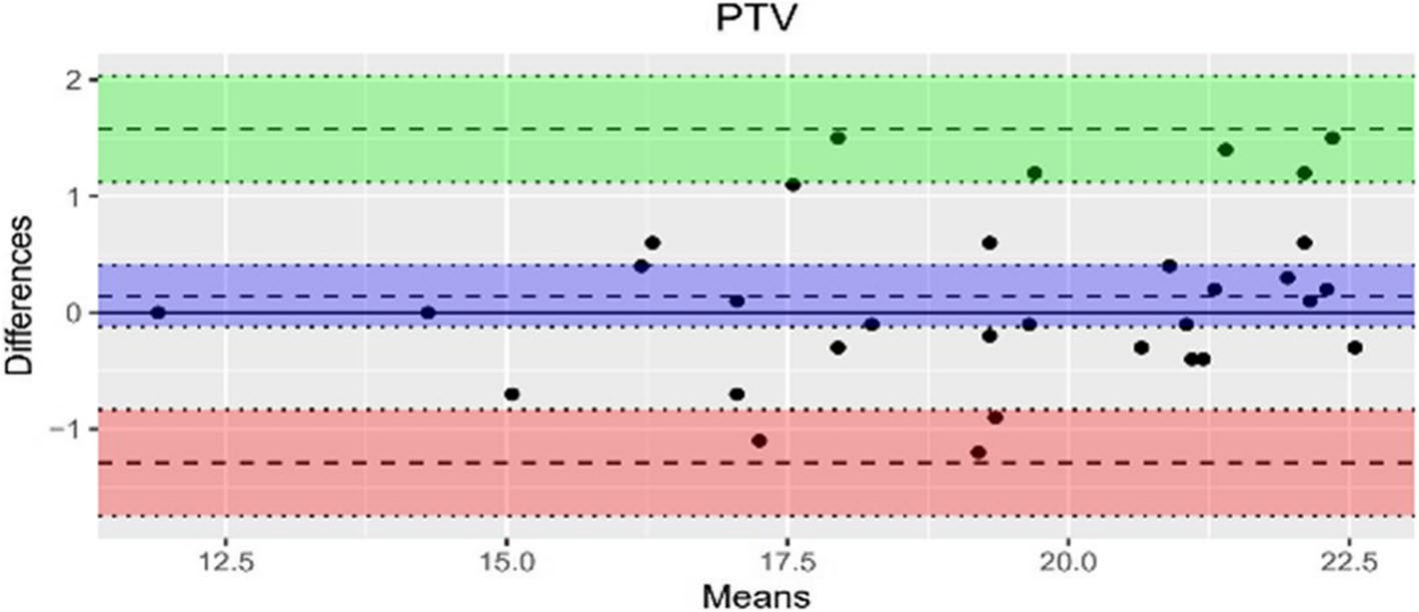
Bland-Altman 6-PTV [mm]

The measurements for the bias revealed a mean difference of -0.034 mm and 0.003 mm. In contrast, the findings for parameter 6-PTV with a mean difference of 0.143 mm show a larger upper and lower measurement range, suggesting a significant degree of variability. However, the results demonstrate a good agreement and reliable results. It is noticeable that the majority of measurements conducted by the examiner fall within the upper and the lower limits as shown in the Bland–Altman diagrams below (Figs. 4, 5 and 6). Moreover, the agreement between the investigations was assessed by using the ICC as listed in Table 3. The average results of the ICC_3_ for inter-rater reliability measured amount to 0.87 mm for the WALA ridge, 0.96 mm for 6-PTV and 0.99 mm for 1-NB.

**Table 3:** Agreement and reliability of repeated measurements (Lb: lower bound, Ub: upper bound)

Type	6-PTV [mm]			WALA ridge [mm]			1-NB [mm]		
	F	Lb	Ub	F	Lb	Ub	F	Lb	Ub
**ICC1**	0.962	0.926	0.981	0.870	0.752	0.934	0.995	0.990	0.997
**ICC2**	0.962	0.926	0.981	0.870	0.752	0.934	0.995	0.990	0.997
**ICC3**	0.962	0.925	0.981	0.870	0.751	0.934	0.995	0.990	0.997
**ICC1k**	0.981	0.961	0.990	0.930	0.859	0.966	0.997	0.995	0.998
**ICC2k**	0.981	0.961	0.990	0.930	0.859	0.966	0.997	0.995	0.998
**ICC3k**	0.981	0.961	0.990	0.930	0.857	0.966	0.997	0.995	0.998

The descriptive statistics are presented in Table 4 using the mean and standard deviation of measurements before and after the orthodontic treatment (T0-T1). Consequently, the measurements of the dental casts show a slight decrease in the post-treatment values. Considering the vertical dimension, the overjet changed by the amount of average 1.51 mm and in the sagittal plane the overbite changed by average 2.73 mm. The cephalometric values include skeletal and dental parameters. For 1-NA and OK1-NSL, a change in inclination of the upper incisors of -2.39° and -1.97°, respectively, was noted (T0-T1). The inclination of the lower anterior teeth changed, with the mean difference being -3.91° for 1-NB and -4.44° for UK1-ML. A larger measurement value was observed for the skeletal sagittal parameter SNB, which amounted 1.68°, while SNA remained unchanged.

**Table 4:** Measurements before and after treatment

Parameter		T0		T1		T0-T1
		mean	SD	mean	SD	mean
**Dental cast**	Overjet [mm]	3.73	1.76	2.22	1.31	1.51
	Overbite [mm]	2.73	2.29	2.06	1.05	0.67
	Lower-Intercanine-distance [mm]	26.5	1.68	26.95	1.46	-0.45
**Lateral cephalogram**	SNA°	79.78	3.34	79.69	3.61	0.09
	SNB°	75.95	3.17	77.6	3.4	-1.68
	ANB°	3.72	2.36	2.1	2.1	1.63
	NSBa°	129.29	4.05	129.74	5,05	-0.44
	ML-NL°	27	5.8	24.4	6.5	2.54
	ML-NSL°	34.55	6.74	32.45	7.19	2.1
	NL-NSL°	7.3	3.5	7.8	3.4	-0.44
	1-NA°	22.98	7.04	25.36	4.08	-2.39
	1-NB°	24.05	6.32	27.96	6.47	-3,91
	OK1-NSL°	102.85	7.94	104.82	5.21	-1,97
	UK1-ML°	93.81	7.72	98.26	8.42	-4,44

The multiple comparison test (ANOVA) showed a statistically significant difference in the measurements of 6-PTV, 1-NB and the WALA ridge (*p* < 0.01) (Tab. 6). The Tukey test was performed to determine the measurement differences between the pre- and the post-treatment values and with regard to the threshold values. Thereby, no statistically significant differences could be identified for the WALA ridge in the measurements of T1-T0 (*p* > 0.05) and a significant difference for T0-G and T1-G (*p* < 0.001). For 1-NB there was a significant difference in all three measurements (T0-G, T1-T0 *p* < 0.05 and T1-G *p* < 0.001). Regarding 6-PTV, no statistically significant difference could be observed in the measurements of T0-G (*p* > 0.05), while there was found a statistically significant difference for T1-G ad T1-T0 (*p* < 0.001). As shown in Table 5, the mean values for 6-PTV at times T0 and T1 were 15.37 mm (SD 2.59) and 20.31 (SD 1.81), respectively. For WALA, 2.56 mm (SD 0.4) was measured at T0 and 2.4 (SD 0.4) at T1. For 1-NB, the two values were 4.23 mm (SD 2.47) and 5.55 mm (SD 2.49), respectively.

**Table 5:** Shapiro–Wilk-Test

	**Parameter**	**n**	**Mean**	**95% CI**		**SD**	**Shapiro–Wilk**
				Lower	Upper		*p*-value
**T0**	6-PTV	32	15.37	11.9	21.4	2.59	0.1424
	WALA	32	2.56	1.75	3.2	0.4	0.7033
	1-NB [mm]	32	4.23	-0.7	8.6	2.47	0.9526
**T1**	6-PTV	32	20.31	16.6	23.1	1.81	0.5341
	WALA	32	2.4	1.65	2.95	0.4	0.8448
	1-NB [mm]	32	5.55	1.4	11.1	2.49	0.3623

In addition to the results described above, Table 5 also shows the inferential statistics of the three parameters measured before and after orthodontic treatment with fixed multibracket appliance, as well as the results of the Shapiro–Wilk test for the 32 subjects.

**Fig. 7:**
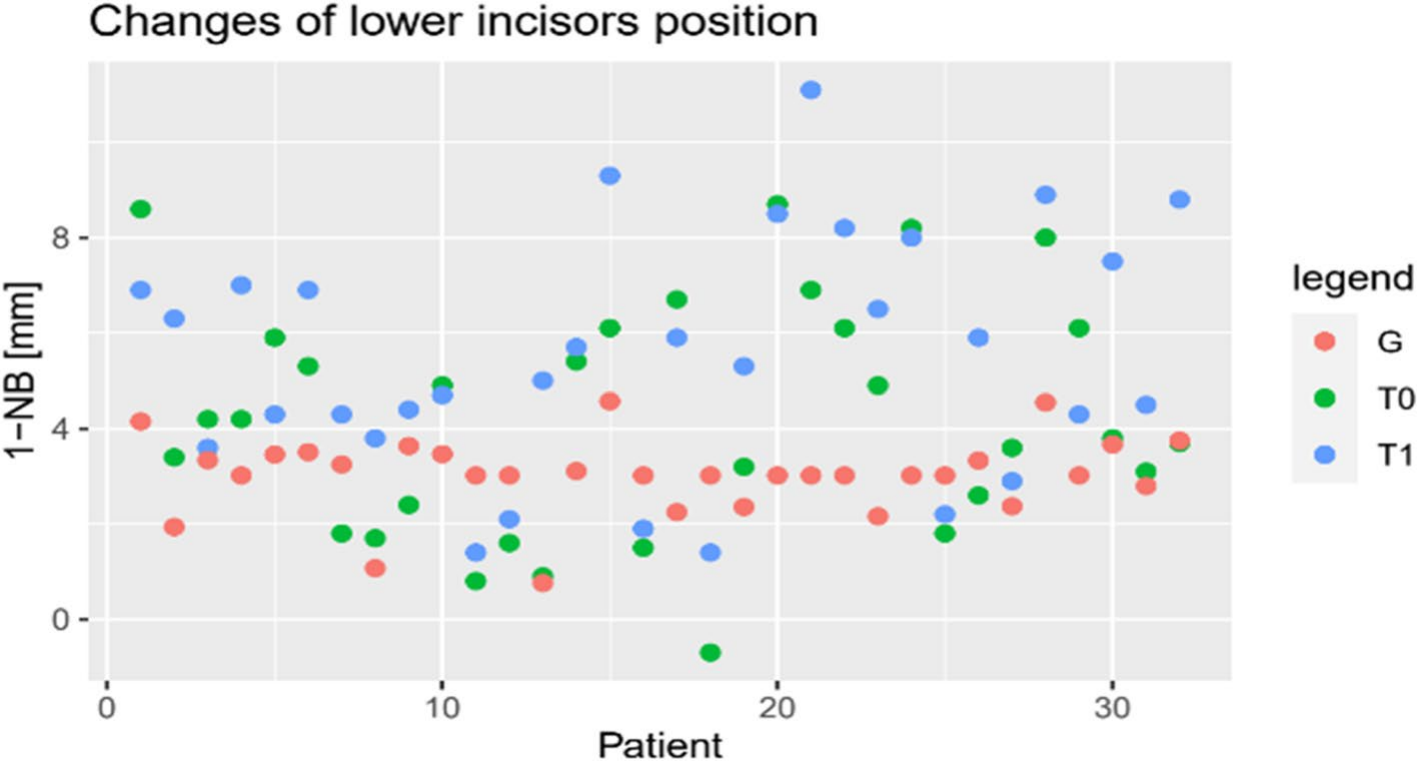
1-NB [mm]

**Fig. 8:**
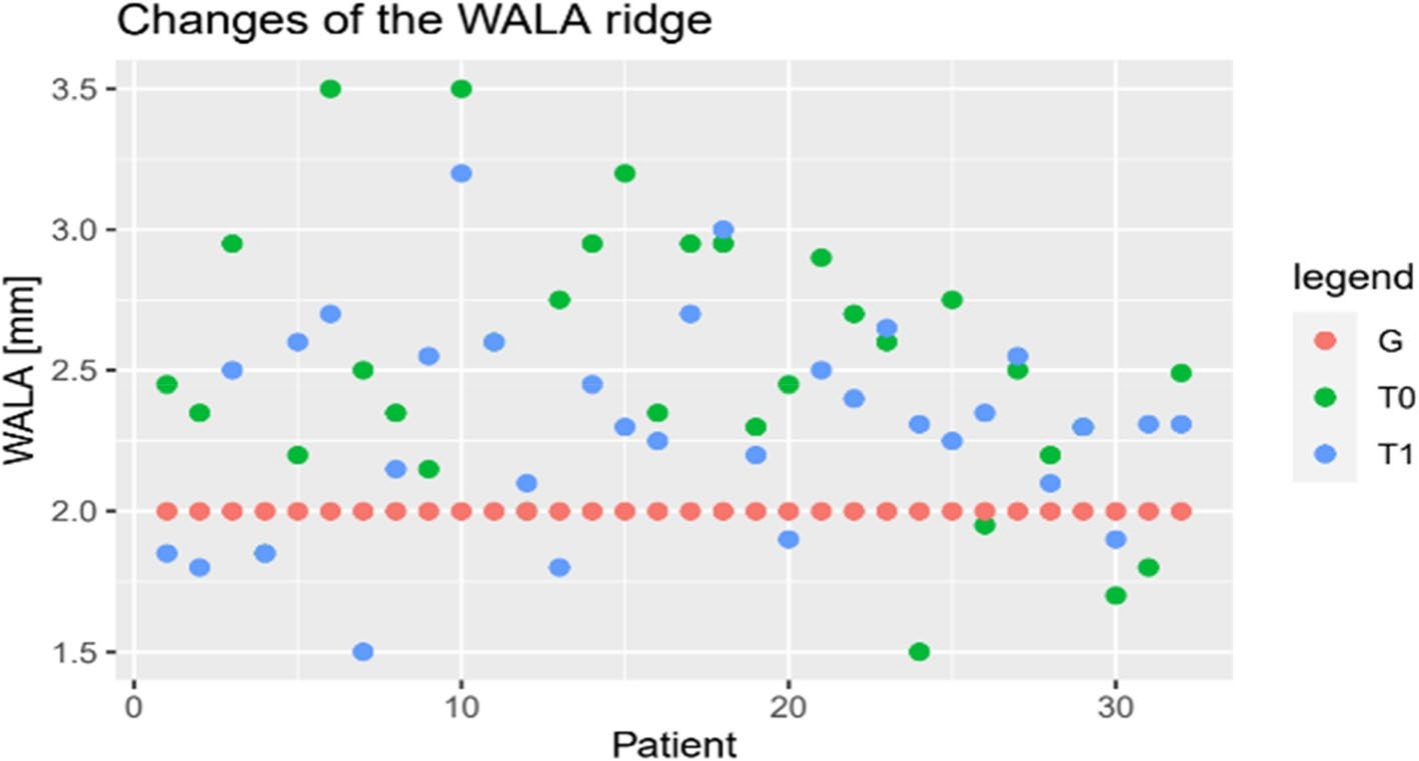
WALA [mm]

**Fig. 9:**
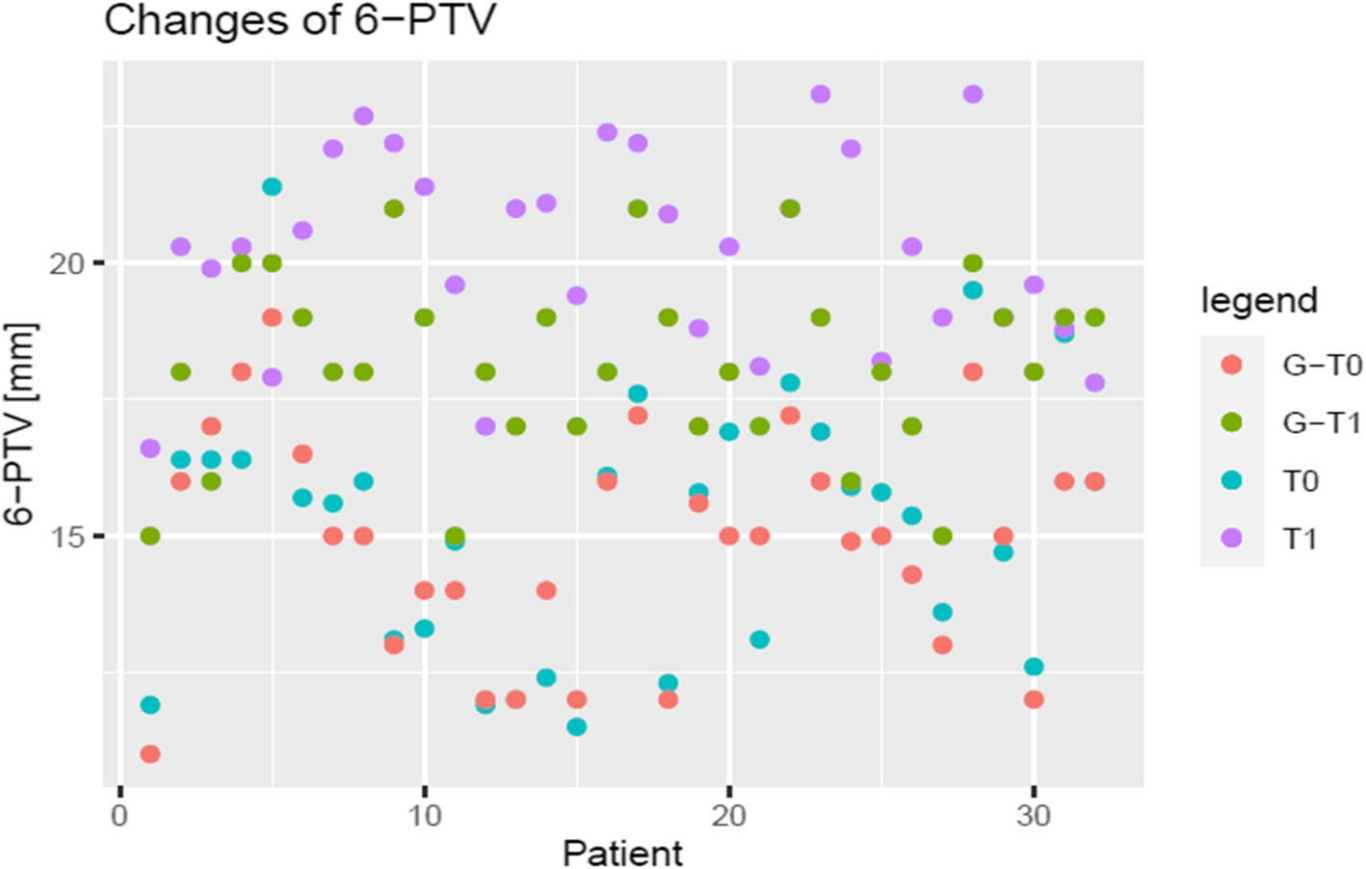
6-PTV [mm]

The assumption of normality was confirmed for all parameters (*p* > 0.05). As demonstrated by the scatter plots (Figs. 7, 8 and 9), all parameters show adjustments resulting from orthodontic treatment. The changes in 6-PTV and 1-NB indicated an increasing trend with respect to the initial measurements. Furthermore, these measurements deviate from the predefined individual limit values. A decrease was observed for the most part in the measured values of the WALA ridge. Besides, it can be stated that the outcomes of the t-test indicate a statistically significant difference in the measurements of all three parameters for T0-T1, T0-G and T1-G (*p* < 0.01).

## Discussion

The primary objective of the present study was to detect a dentoskeletal frame within which orthodontic tooth movement can be implemented taking into account the anatomical limits. In addition, the feasibility of certain reference values should be examined more closely. Therefore, we used 1-NB as anterior sagittal border for the lower incisors. For first maxillary molars, 6-PTV represented the sagittal posterior limit and the WALA ridge limited the range of dental movement of lower first molars in the transverse dimension. For this purpose, 1-NB and 6-PTV were measured on lateral cephalograms, while the WALA ridge was evaluated on digital dental casts in the software OnyxCeph^3^™. The selected data was statistically analyzed before and after orthodontic treatment with specified values. For 1-NB, the individually calculated reference value was applied according to the regression equation established by Segner and Hasund [12]. In addition, the calculation formula developed by Ricketts^3^ was used to assess the individual values for 6-PTV and the WALA ridge introduced by Andrews [13] was applied with an average of 2 mm for the first molars.

At the outset, it is important to mention that the random error according to Bland–Altman was calculated using all measurements of the parameters of the 32 patients (T0) within a 30-day interval. For the measurements of 6-PTV, the results illustrate a limit of agreement range between -1.29 and 1.58 with a bias of standard deviation of 0.73 and a mean difference of bias of 0.14. For the WALA ridge, the range of agreement lies between -0.44 and 0.45 with a bias standard deviation of 0.23 and an average difference of bias of 0.003. Furthermore, the statistical analysis for 1-NB based on Bland–Altman yields a limit of agreement of -0.5 to 0.44, a bias standard deviation of 0.24 and a mean difference of bias of -0.03. All measurements show a good level of agreement, which is confirmed by the CI (95%).

Moreover, it is important to note that ICC values greater than 0.80, according to the literature, indicate excellent reliability [28, 29]. The ICC_3_ was selected for the present study because the measurements were carried out twice by one single examiner. The high-level consistency of all measurements was confirmed with ICC values higher than 0.80 (Tables 2). This suggests a remarkable reliability and consistency in the measurements performed by the main investigator. The intra-rater reliability and the inter-rater reliability were further investigated by Sayinsu et al. [30] for some manually measured cephalometric values and compared to measurements made with the Dolphin Imaging Software 9.0 (Los Angeles, California, USA). A very good reliability of ≥ 0.90 was shown. Furthermore, the measurement accuracy provides a high correlation between the conventional method and the digitized method. In order to emphasize the accuracy of measurements in digital models, an additional study was identified that addressed the statistical analysis [31]. The ICCs were 0.84 ± 0.15 (intra-examiner) and 0.80 ± 0.19 (inter-examiner) with an average difference of 0.23 ± 0.14 and 0.24 ± 0.11. The results are similar to our results and thus demonstrate and confirm a high reproducibility and precision with digitally analyzed models.

The pre- and post-therapeutical cephalometric analysis revealed a slight sagittal development of the mandible (SNB: T0: 75.95°, T1: 77.6°; ANB: T0: 3.72°, T1: 2.1°) while the maxilla remained unchanged. The changes for the inclination of the maxillary anterior teeth are presented by the angles 1-NA and OK1-NSL, which amount to -2.39° and -1.97° and for the lower incisors the mean difference for 1-NB was -3.91° and for UK1-ML -4.44° (T0-T1). These variations imply a protrusion of all anterior teeth. On further examination the transverse dimension of the dental casts, a marginal increase in the lower intercanine distance was noted (T0-T1: -0.45 mm), but this is classified as statistically not significant. In the vertical plane the overbite decreased by 0.67 mm and in the sagittal dimension the overjet declined by 1.51 mm.

Based on the results of the ANOVA test, the differences in all measurements were statistically significant (G-T1: *p* < 0.05) (Table 6). This suggests that all parameters have changed as a result of the orthodontic treatment. The WALA ridge showed the successful alignment of the molars, while the measurements of 6-PTV indicate a mesial movement of the posterior teeth. However, with regard to 6-PTV, it is unclear whether the movement is due to tipping, translation or anchorage loss. This observation requires further research by performing measurements using three-dimensional radiographs like a Come Beam Computed Tomography (CBCT) before and after treatment. The changes in the 1-NB indicate protrusion of the anterior teeth exceeding the individualized reference values, as illustrated in Fig. 7. This fact emphasizes the relevance and the necessity of sufficient means of anchorage during orthodontic treatment.

**Table 6:** ANOVA Analysis Results (*sign.)

Parameter	Sum of squares	df	Mean squares	F	*p*
6-PTV	657.4	93	328.7	60.21	< 2e-16 *
WALA	3.937	93	1.968	16.14	9.63e-07 *
1-NBmm	102.0	93	51.02	11.81	2.69e-05 *

The results of the t-test indicate that the differences between the measurements (T0-T1, T0-G and T1-G) are unlikely to be attributed to random chance and are therefore statistically significant (*p* < 0.01). Moreover, through the measurements (T1) it can be concluded that all three parameters exceed the reference values (G) drawn in Figs. 7, 8 and 9. This raises the question of the long-term stability of treatment results.

In the literature, a variety of studies can be identified that investigated those aspects in more detail. Ramos et al. [32] and Hasund et al. [33] underlined the relevance as well as the benefit of the Steiner analysis. They also mentioned that with vertical growth pattern, the value of 1-NB should be slightly increased at the end of orthodontic treatment, while with horizontal growth pattern, it should be reduced in order to obtain a stable treatment result.

Navarro et al. [34] selected ninety patients with Class I and Class II malocclusions for their study, with subgroups divided into horizontal, vertical and balanced growth patterns. The changes of 1-NB before and after treatment considering the values proposed by the Steiner analysis were examined thoroughly. The study results show statistically significant differences between the reference values and the actual treatment results among the participants with vertical growth pattern and those showing a balanced growth pattern, whereby the reference values were not attained. An analogous behavior was witnessed by Farret [35]. He noted a proclivity for not achieving the proposed values for 1-NB as the final results show a mean difference of 1.36 mm.

However, the above-mentioned investigations were conducted taking into consideration the Steiner analysis using generalized average values as reference. Our investigation was carried out considering individually calculated threshold values based on regression equation by Segner and Hasund [12]. The use of individual objectives enables more precise examinations. Nevertheless, a significant weakness of our study lies in the disregard of the growth pattern and the type of malocclusion, which could have helped clarify a tendency depending on the presence of a certain malocclusion.

As reported by Andrews [36] in 2015, a mean value should be determined for the distance between the WALA ridge and the core line of FA points. The ideal value for a normal occlusion should be 2 mm in the position of the first molars. Kong-Zarate et al. [17] and Trivino et al. [37] confirmed these findings with similar outcomes (2.12 mm and 2.21 mm). In order to carry out more detailed investigations, Mahalakshmi et al. [38] measured dental casts of 20 patients aged 19–35 years with Angle Class I, Class II and Class III. The statistically significant results are at 1.87 mm ± 0.52, 1.67 mm ± 0.45 and 1.51 mm ± 32 (*p* < 0.01), thus proving to be close to the measurements of the previously reported studies. Despite the fact that Mahalakshmis’ means are smaller in the groups of Class II and Class III patients and suggest a lingual tipping of the posterior teeth.

A few years later Esteves et al. [39] analyzed 60 dental casts of patients with Class I malocclusion and divided them into two groups. One group was treated with conventional brackets and the other group was treated with a passive self-ligating bracket system. In both groups an increase of the WALA ridge was shown. This was due to the horizontal alignment of the posterior teeth. In the group treated with the self-ligating brackets a slightly greater increase could be obtained (*p* < 0.05). In the present study the measurements for the WALA ridge at T0 and T1 amount to 2.56 mm ± 0.40 and 2.40 mm ± 0.40, respectively, and therefore, presented an acceptable treatment result. Furthermore, in the literature the three studies by Ball et al. [40], Gupta et al. [41] and Ronay et al. [42] were found to fit this topic. They prove that the reference value of 2 mm set by Andrews for the WALA ridge represents a realistic treatment goal with respect to the anatomical limitation. Based on the pre- and post-therapeutic dental measurements in treatment with fixed appliances, the investigators found a post-treatment WALA ridge of 2.6 mm ± 0.5, 2.38 mm and 2.62 mm ± 0.22 for the first molars. However, further investigation is needed to identify whether the tooth movement is about purely bodily movement or if it takes place as a result of dental tipping.

**Fig. 10:**
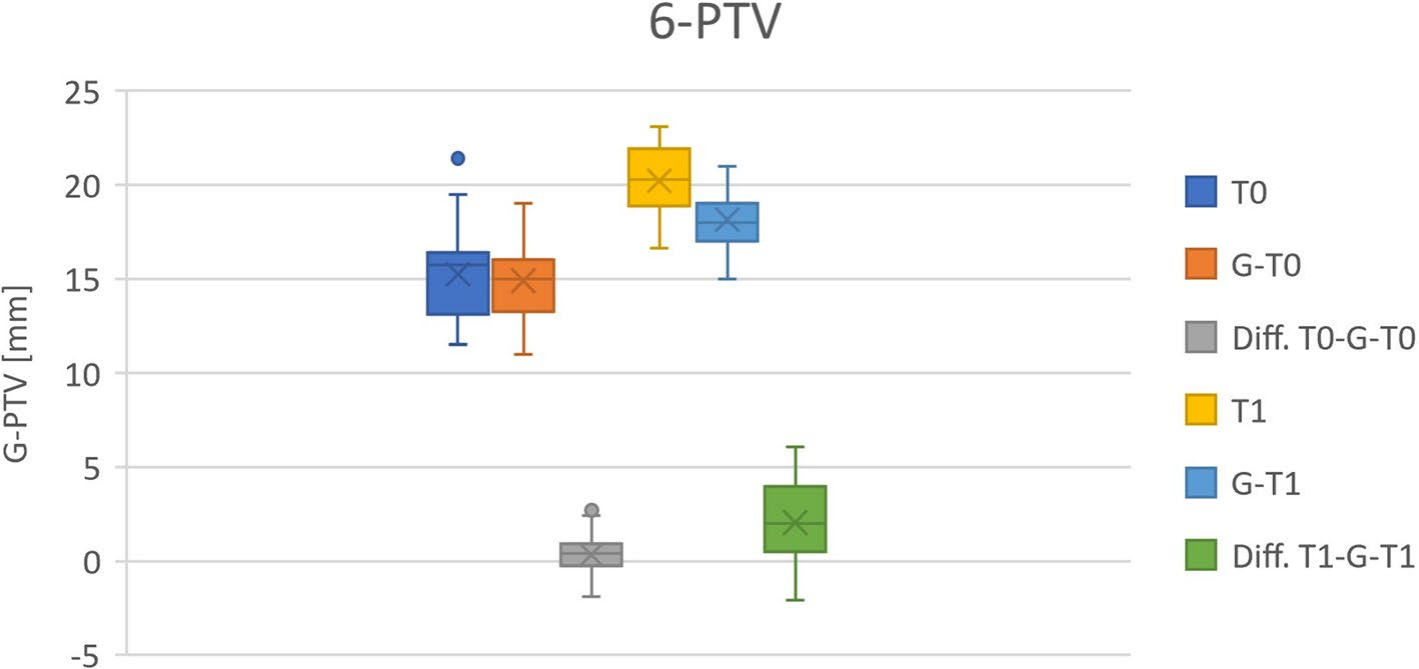
6-PTV [mm]

To sum up, it is important to mention that the available retromolar space plays a remarkable role in orthodontic treatment planning. Hereby, the distance between the distal approximal edge of the upper first molars along the occlusal plane and the intersection of the pterygoid vertical (PTV) was measured (6-PTV) [3, 4]. In the present study, it was shown that the molars moved forward despite orthodontic treatment being performed. This was determined using Turkey test, where the distance of 6-PTV changed significantly (T1-G: *p* < 0.01) (Figs. 9 and 10). Furthermore, the measurements before and after the treatment deviate from the reference values calculated by the formula developed by Ricketts^3^ (T0: 0.34 mm, T1: 2.06 mm). This manifests the obtainment of a more mesial position. Therefore, the applicability as well as the relevance of the formula are questionable. An additional hypothesis is that the posterior teeth moved mesially due to anchorage loss or the early loss of primary teeth. Finally, another aspect that casting doubt on the plausibility of Rickett`s age-related formula is the fact that participants may have grown more than expected.

These findings are validated by the study of Rajesh et al. [43] and Su et al. [44]. Based on both investigations a mean anchorage loss of 1.8-2 mm and 2.37 ± 1.72 mm was discovered during the initial phase of levelling. In cases where cinch backs are placed just behind the last bracket of the posterior teeth, anterior movement of the incisors is minimized. It is important to note that this also requires additional means of anchoring in the posterior region in order to prevent mesial movement of the posterior teeth [45, 46].

As creating Virtual Treatment Objectives (VTO) is a common part of the orthodontic treatment planning, it is important to know the biological limitations. When employing a software, teeth may be moved in various dimensions, whereby one should adhere the biological limits without exceeding them. Therefore, it is essential to establish a connection between the dental cast and the lateral cephalogram. This allows for beneficial utilization of the lateral cephalogram in planning tooth movements as well as anchorage planning.

### Limitation

The retrospective study design contains some deficiencies, as some important aspects were not considered in the investigation. These aspects include, for example, patient compliance, oral hygiene or growth pattern. In addition, the existence of a possible pre-treatment with myofunctional appliance and the comparison to an untreated control group were also not incorporated. The sample size eventually proved to be sufficient and only slight heterogeneity was found. The patients were consecutively selected to ensure a more representative sample, as the selection process is thus more random and less biased. However, this approach also entails constraints, as it is not possible to delineate the potential challenges related to biological limitations specific to various malocclusions.

## Conclusion

Considering the previous discussion, the following conclusion was drawn:

- The measurements of the three parameters (6-PTV, 1-NB, WALA ridge) changed as part of orthodontic treatment.
- The threshold values determined are feasible but not entirely manageable without exceeding the biological limits.
- The examined parameters provide a useful guidance defining the biological limits of the sagittal posterior, sagittal anterior and transverse dimensions.

Clinical relevance:

- As first step, the newly acquired knowledge from the present study offers the opportunity of ensuring orthodontic VTO within biological limits.
- The study also showed that the lateral cephalograms are indispensable and serve as an important source of dental and skeletal information. Lateral cephalograms should also be incorporated into the virtual treatment setups.

## Data Availability

We confirm that the data supporting the findings of this study are available within the article and its supplementary materials.
