# Correction: Orthodontic treatment and biological limits: a retrospective clinical trial

**DOI:** 10.1186/s13005-023-00405-x

**Published:** 2024-01-08

**Authors:** Niki Nikoleta Tabancis, Karl‑Friedrich Krey, Franka Stahl, Valeria Behnke, Anja Ratzmann

**Affiliations:** 1 Department of Orthodontics and Craniofacial Orthopaedics, University Medicine of Greifswald, Walther‑Rathenau‑Strase 42a, 17489 Greifswald, Germany; 2 Department of Orthodontics and Craniofacial Orthopaedics, University Medicine of Rostock, Strempelstrase 13, 18057 Rostock, Germany

**Correction: *Head Face Med *19**, **53 (2023)**

10.1186/s13005-023-00399-6

Following publication of the original article [1], the author identified a mistake regarding the affiliation of Professor Karl-Friedrich Krey and Professor Franka Stahl. In the publication it is printed the opposite.

Therefore, the author has requested to correct the following issues:

Incorrect: Karl-Friedrich Krey − 2 Department of Orthodontics and Craniofacial Orthopaedics, University Medicine of Rostock, Strempelstraße 13, 18,057 Rostock, Germany; Franka Stahl − 1 Department of Orthodontics and Craniofacial Orthopaedics, University Medicine of Greifswald, Walther Rathenau Straße 42a, 17,489 Greifswald, Germany.

Correct: Karl-Friedrich Krey − 1 Department of Orthodontics and Craniofacial Orthopaedics, University Medicine of Greifswald, Walther Rathenau Straße 42a, 17,489 Greifswald, Germany; Franka Stahl − 2 Department of Orthodontics and Craniofacial Orthopaedics, University Medicine of Rostock, Strempelstraße 13, 18,057 Rostock, Germany.

The original article has been corrected.
